# The use of multilevel emotion regulation strategies in the context of critical public events: the more the better?

**DOI:** 10.3389/fpsyg.2024.1403308

**Published:** 2024-07-15

**Authors:** Leling Zhu, Jiemin Yang, Jiajin Yuan

**Affiliations:** ^1^Institute of Brain and Psychological Science, Sichuan Normal University, Chengdu, China; ^2^Sichuan Key Laboratory of Psychology and Behavior of Discipline Inspection and Supervision, Sichuan Normal University, Chengdu, China

**Keywords:** mental health, emotion regulation strategies, critical social events, social media use, longitudinal study

## Abstract

Critical public events, like COVID-19, significantly impact individuals’ emotional and mental health. People tend to use multi-level emotion regulation strategies (intrapersonal, interpersonal and hyper-personal) to cope with these events, resulting in various strategy profiles. However, few studies have examined ER strategies from a multilevel perspective. Therefore, this study examines the use of multi-level strategies during COVID-19, and evaluates the effectiveness of these strategies, with a particular interest in identifying strategy profiles promoting mental health. We conducted a two-wave study (an interval of 1 week) using online questionnaires during COVID-19, with an initial sample of 1,189 participants and 895 samples completing the surveys across the two waves. Cross-lagged analysis indicated that experiential avoidance was reciprocally positively related to negative emotions while perspective-taking and humorous-meme-saving were reciprocally positively related to life satisfaction or positive emotions over time. Cluster analysis suggested that there were 9 different profiles which scored differently on mental health indicators. Specifically, the use of multi-level strategies tended to be associated with greater positive emotions and life satisfaction while with lower negative emotions and loneliness. This study revealed that the use of multi-level strategies plays a protective role in mental health when facing critical public events. These findings expanded our understanding of how multilevel emotion regulation strategies impact mental health during critical public events and identify protective profiles for mental health.

## 1 Introduction

Recent years have witnessed several critical public events, which always significantly impact individuals’ emotional and mental health. COVID-19, as a typical public health emergency, has brought people an increase in negative experiences including fear, anxiety, and loneliness, and a decrease in positive emotions and well-being during the pandemic (O'Sullivan et al., 2021). Considering that similar critical events may arise in the future, COVID-19 may provide a good opportunity for us to investigate the protective factors that can enhance mental health in these challenging circumstances.

Prior studies have demonstrated that emotion regulation is crucial for maintaining psychological well-being (John and Gross, 2004; Aldao et al., 2010). However, whether emotion regulation promotes psychological adjustment to critical public events depends on flexible strategy use (Bonanno and Burton, 2013). That is, to better adjust to critical public events, individuals should consider contextual demands and select appropriate strategies. The more the chosen strategy adapts to the situation, the more effectively it can help individuals achieve their emotion regulation goals, resulting in higher utility (Aldao et al., 2010; Bonanno and Burton, 2013). Moreover, possessing the capability to select and adjust strategies according to the circumstances is indicative of one’s psychological well-being. Research indicates that psychological flexibility is a fundamental aspect of promoting healthy personal and social functioning, while inflexibility is associated with an increased risk for psychopathology such as depression and anxiety (Kashdan and Rottenberg, 2010; Chen and Bonanno, 2021). Therefore, the relationships between mental health and ER strategies are likely to be bidirectional. The first aim of this study is to re-examine the relationship between ER strategies and mental health during the pandemic.

Specifically, one component of ER flexibility is an individual’s ER repertoire, which refers to the range of different ER strategies an individual utilizes across situations (Bonanno and Burton, 2013). To better cope with the intense and prolonged negative emotions caused by critical public events, individuals may endeavor to use multiple emotion regulation strategies, rather than the one or two strategies offered in laboratory settings to change their current affective state (Cheng et al., 2014; Grommisch et al., 2020). As we reflect on the evolution of emotion research, we can observe that in its early stages, researchers primarily concentrated on the influence of individuals in determining their emotions, the timing of these emotions, and their expression (Gross, 1998). Over time, researchers began to recognize that emotions are typically experienced, expressed, and controlled within the context of social interactions, leading to the integration of emotion regulation into a framework of interpersonal regulation (Zaki and Williams, 2013). With the increasing prevalence of social media in daily life and its pervasive impact on various aspects of life, researchers have begun to explore emotion regulation within the context of social networks (Beaudoin and Hong, 2021; Shao et al., 2021). It appears that emotion regulation occurs within various levels of relationships; however, there has been limited research on emotion regulation strategies from a comprehensive and multilevel standpoint. Therefore, this study delved into emotion regulation through a multilevel perspective to enhance our understanding of this process.

Concerning intrapersonal-level strategies, cognitive reappraisal and experiential avoidance are two common strategies when facing uncontrollable situations like the pandemic (Tamir et al., 2007; Troy et al., 2013). Cognitive reappraisal, which involves reconstructing the emotion-eliciting situation differently (John and Gross, 2004), is considered particularly adaptive and useful (Troy et al., 2013). Experiential avoidance is defined as a rigid pattern of attempting to avoid or escape unwanted internal experiences such as distressing thoughts, emotions, or physical sensations (Cao et al., 2013). Although it can be useful for avoiding threats, it should not be regularly employed (Tamir et al., 2007; Akbari et al., 2022). Interpersonal emotion regulation is one of the main sources that individuals receive social support, which is important for buffering negative outcomes during shared adverse experiences (Levy-Gigi and Shamay-Tsoory, 2017; Harp and Neta, 2023). Hofmann and his colleagues classified interpersonal emotion regulation strategies into four types: (1) enhancing positive affect, describing the tendency to seek out others to increase feelings of happiness and joy; (2) perspective taking, which involves using others to remind oneself not to worry and that others have it worse; (3) soothing, which consists of seeking out others for comfort and sympathy; and (4) social modeling, which involves looking to others to see how they might cope with a given situation (Hofmann et al., 2016). Researchers have suggested that overreliance on interpersonal emotion regulation could compromise individuals’ emotional control abilities (Hofmann et al., 2016).

Hyper-personal emotion regulation pertains to the use of digital technology to manage emotions and moods such as posting and retweeting emotional content, which provides more strategy choices that can be deployed with less effort (Smith et al., 2022). However, it should be noted that individuals who use social media may be susceptible to being affected by other people’s emotional expressions online, which can potentially lead to feelings of anxiety or emotional exhaustion (Prikhidko et al., 2020; Shao et al., 2021). In summary, various levels of strategies present their advantages and disadvantages, therefore leveraging a combination of these strategies may result in more favorable outcomes. During COVID-19, people have had to reduce their offline interactions and rely more on social media to reduce the chances of infection (Vall-Roque et al., 2021), which enabled the widespread use of hyper-personal emotion regulation. Researchers noticed that pandemic-related jokes and memes rapidly circulate on social media (Skorka et al., 2022), which fell under the category of hyper-personal emotion regulation. Numerous studies suggest that humor is an instinctive coping mechanism that has a wide range of significant positive effects on psychological health (Berger et al., 2021). By posting and/or retweeting these humorous materials, individuals may find it easier to manage challenges and setbacks by eliciting positive emotions (Rime et al., 2020). In other words, humorous content shared on social media allows people to utilize and communicate humor through their social network, which plays a protective role during critical public events.

In summary, people tend to use multiple emotion regulation strategies to regulate their emotions in daily life, which is the combination of different levels of strategy, resulting in different profiles of strategies (Grommisch et al., 2020; Rottweiler et al., 2023). Every strategy has its advantages and disadvantages in certain contexts, and the combination of different levels of strategies may yield different outcomes. However, although social media has gradually become part of people’s daily lives and an important venue for emotion regulation (Blumberg et al., 2016), few studies include hyper-interpersonal level strategies in emotion regulation research and discussed the use and mental consequences of all three levels of emotion regulation strategies simultaneously (Altan-Atalay and Ray-Yol, 2021; Shao et al., 2021; Rottweiler et al., 2023). Hence, it is crucial to examine the emotion regulation strategies of individuals at all three levels and clarify the connection between these strategies and mental well-being. Therefore, we collected two waves of longitudinal data and conducted cross-lagged analysis to examine the relationship between all three levels of emotion regulation strategies and mental health. Cluster analysis was used to identify any profiles of emotion regulation strategies. In this study, mental health was measured by (1) emotion, which are targets of emotion regulation; (2) loneliness, an unpleasant experience or state that has been shown to contribute to a range of physical and mental health issues (Cacioppo et al., 2015). It has been particularly prevalent during the COVID-19 pandemic (O'Sullivan et al., 2021); and (3) life satisfaction, which refers to a cognitive and global evaluation of the quality of one’s life (Pavot and Diener, 2008). This study may increase our understanding of how multilevel emotion regulation strategies predict mental health during critical public events and identify the profiles that are protective for mental health.

## 2 Methods

### 2.1 Participants

We conducted a two-wave study (an interval of 1 week, during which the control measures remained the same) using an online questionnaire during the epidemic. To match participants’ data of the two measuring points of the study, participants were instructed to enter several pieces of personal information consisting of (1) real name, (2) phone number, (3) social media accounts (QQ and WeChat), and (4) Alipay accounts (channels for payment of partition fees). Informed consent was obtained from all participants, and all respondents were informed that all their data would be used for scientific purposes only and will be kept confidential. They could terminate participation at any time during the study. The initial sample included 1,189 participants, with 859 participants completing the surveys in both waves. Participants who only completed the survey at Time 1 did not significantly differ from the longitudinal participants in terms of demographic and study variables. At Time 1, 71% of the 859 participants (45% male) were in the low-risk area,^1^ and at Time 2, 78% of participants (46% male) were in the low-risk area. The study received ethical approval from the host university to launch the study.

### 2.2 Measures

#### 2.2.1 Emotion assessment

Emotions are measured by the 18-item Chinese revision of the Positive and Negative Affect Scale, of which 9 items assess positive affect (Cronbach α_T1_ = 0.937; Cronbach α_T2_ = 0.952), and the rest assess negative affect (Cronbach α_T1_ = 0.897; Cronbach α_T2_ = 0.916), both are presented on a 5-point Likert scale (Qiu et al., 2008).

#### 2.2.2 Mental health

We adopt the 5-item Chinese revision of the Satisfaction with Life Scale (Xiong and Xu, 2009), items are presented on a 7-point Likert scale (T1: Cronbach α = 0.92; T2: Cronbach α = 0.92). Loneliness is measured by the 6-item Chinese revision of the short form of the UCLA Loneliness Scale (Cronbach α_T1_ = 0.876; Cronbach α_T2_ = 0.877), items are presented on a 4-point Likert-type scale (Zhou et al., 2012).

#### 2.2.3 Emotion regulation strategies

Cognitive reappraisal is measured by the reappraisal subscale of the Chinese version of the Emotion Regulation Scale (Wang et al., 2007), 6 items are presented on a 7-point Likert-type scale (Cronbach α_T1_ = 0.822; Cronbach α_T2_ = 0.839). Experiential avoidance is measured by the Chinese version of the Acceptance and Action Questionnaire-Second Edition (Cao et al., 2013), 7 items are presented on a 7-point Likert scale (Cronbach α_T1_ = 0.914; Cronbach α_T2_ = 0.924).

Interpersonal emotion regulation strategies are measured by the 20-item Chinese version of the Interpersonal Emotion Regulation Questionnaire (Chen et al., 2021), items are presented on a 5-point Likert scale (Cronbach α_T1_ = 0.921; Cronbach α_T2_ = 0.934).

Hyper-personal emotion regulation strategies are reflected in the willingness to retweet and the saving behavior of humorous memes. Saving behavior was chosen because people who saved the memes from the questionnaire were more likely to retweet them to others. First, we searched and downloaded 104 memes from the Internet. Second, we recruited 170 participants to rate the pandemic relevance and fun of these memes. Finally, we collected 10 memes for formal experiments. Willingness to retweet was measured by asking participants how likely they were to retweet the humorous memes (ranging from 1 to 5; Cronbach α_T1_ = 0.884; Cronbach α_T2_ = 0.918); saving behavior was measured by asking participants whether they saved the humorous memes (yes or no; Cronbach α_T1_ = 0.865; Cronbach α_T2_ = 0.893).

### 2.3 CFA

Confirmatory factor analysis (CFA) was conducted in Mplus 8.3 (Muthén and Muthén, 2017) to test the measurement models for the present study. All the results in two waves revealed a good model fit for all scales (χ^2^/df < 5.5, CFIs>0.9, RMSEAs<0.08, SRMRs<0.08), indicating that all scales had sound measurement properties.

### 2.4 Data analysis

We computed a cross-lagged structural model using Mplus 8.3. The default estimation method of maximum likelihood was used. To study the adequacy of the estimated model, we used χ^2^/df, the RMSEA, the CFI, the TLI, and the SRMR. We employed Mclust, a popular R package for model-based clustering, classification, and density estimation based on finite Gaussian mixture modeling (Scrucca et al., 2016), to develop profiles of emotion regulation strategies based on the data collected at T1 and T2. This method does not require the researcher to make assumptions about the shape and size of the clusters that are difficult to justify. In the model-based clustering approach, each component of a finite mixture density is usually associated with a group or cluster, and in the model, it is assumed that the mixture components share the same orientation matrix. The number of mixing components and the covariance parameterization are selected using the Bayesian Information Criterion (Scrucca et al., 2016).

## 3 Results

### 3.1 Common method bias

The data collected in this study were controlled for common method bias through anonymous collection and inverse item scoring. We used Harman’s single-factor test to check for common method bias. and the result showed that the first factor only explained 22.16% at T1 and 26.6% at T2 of the variance, below 40%, which means that common method bias did not threaten the interpretation of our results.

### 3.2 Descriptive and correlation

Descriptive statistics of the variables are shown in Table 1. We employed paired sample T-test analysis of variance and found that positive emotion and life satisfaction were improved in T2, and negative emotion and loneliness were decreased in T2. Additionally, there were also changes in emotion regulation strategies except cognitive reappraisal, enhancing positive emotion, and soothing. Specifically, the use of experiential avoidance and retweeting willingness were decreased in T2, and the use of rest strategies was increased in T2. These results suggest that participants’ mental health improved over time, and emotion regulation was a dynamically changing process.

**Table 1:** Descriptive statistics and comparison of variables.

Variables	T1		T2		*t*	*p*
	M	SD	M	SD		
Negative emotion	2	0.82	1.93	0.83	3.286	0.001
Positive emotion	2.95	1.03	3.18	1.06	−8.616	< 0.001
Life satisfaction	22.19	7.3	22.94	7	−4.655	< 0.001
Loneliness	11.81	3.95	11.59	3.79	2.269	0.024
Cognitive reappraisal	32.58	4.91	32.45	4.84	0.91	0.363
Experiential avoidance	24.89	9.17	24.1	9.12	3.598	< 0.001
Enhancing positive affect	19.86	3.49	19.82	3.34	0.449	0.654
Perspective taking	18.21	3.31	18.59	3.25	−4.152	< 0.001
Soothing	19.06	3.91	19.14	3.73	−0.797	0.426
Social modeling	19.38	3.17	19.56	3.02	−2.095	0.036
Retweeting willingness	2.85	0.97	2.76	1.07	4.017	< 0.001
Save behavior	0.33	0.29	0.36	0.34	−4.903	< 0.001

Results of correlative analysis (see Supplementary Table S1) showed that all strategies except experiential avoidance (AAQ) positively related to positive emotion and life satisfaction, and negatively related to negative emotion and loneliness. Conversely, AAQ exhibited a negative correlation with positive emotion and life satisfaction and a positive correlation with negative emotion and loneliness. In addition, the correlation between different strategies was also significant.

### 3.3 Cross lag analysis

In cross-lag analysis, we controlled for the effects of gender, age, and risk level at two measure points. The cross-lagged model fitted the data well, with χ^2^/df = 4.79, *p* < 0.001, TLI = 0.911, CFI = 0.972, RMSEA = 0.065 (95%CI = [0.059, 0.072]), SRMR = 0.069. Standardized path coefficients are presented in Table 2 (Auto-regressive paths are significant for all variables, *p*s < 0.001. For brevity, auto-regressive paths, control variables paths, and insignificant coefficients are not presented in the table).

**Table 2:** Path coefficients.

Parameter		Estimate	S.E.	Est./S.E.	*p*-value
T1RE→	T2PA	0.098	0.025	3.865	< 0.001
T1AAQ→	T2NA	0.162	0.029	5.558	< 0.001
	T2PA	−0.095	0.025	−3.798	< 0.001
	T2UCLA	0.141	0.028	5.082	< 0.001
	T2SWLS	−0.153	0.023	−6.779	< 0.001
T1PT→	T2SWLS	0.054	0.027	1.993	0.046
T1SAVE→	T2PA	0.067	0.027	2.474	0.013
	T2UCLA	−0.092	0.035	−2.606	0.009
T1NA→	T2AAQ	0.114	0.024	4.655	< 0.001
	T2SM	0.073	0.028	2.663	0.008
T1PA→	T2AAQ	−0.047	0.024	−2.013	0.044
	T2PT	0.071	0.036	1.980	0.048
	T2S	0.114	0.033	3.475	0.001
	T2WILL	0.068	0.029	2.304	0.021
	T2SAVE	0.109	0.027	4.087	< 0.001
T1SWLS→	T2EP	0.120	0.034	3.555	< 0.001
	T2PT	0.106	0.038	2.810	0.005
	T2SM	0.094	0.037	2.563	0.010
	T2WILL	0.063	0.030	2.119	0.034
T1UCLA→	T2EP	−0.056	0.028	−2.027	0.043
	T2S	−0.06	0.029	−2.082	0.037
	T2SM	−0.110	0.031	−3.579	< 0.001

*p < 0.05, **p < 0.01, ***p < 0.001. NA, negative emotion; PA, positive emotion; SWLS, life satisfaction; UCLA, loneliness; RE, cognitive reappraisal; AAQ, experiential avoidance; EP, enhancing positive affect; PT, perspective taking; S, soothing; SM, social modeling; WILL, retweeting willingness; SAVE, save behavior.

The use of cognitive reappraisal at T1 was positively related to positive emotion at T2. The use of experiential avoidance was reciprocally positively related to negative emotions over time, perspective-taking was reciprocally positively related to life satisfaction over time, and the saving behavior of humorous memes was reciprocally positively related to positive emotions over time. The experiential avoidance at T1 was also positively related to loneliness at T2, and negatively related to positive emotion and life satisfaction at T2. The saving behavior of humorous memes at T1 was also negatively related to loneliness at T2. These results may indicate that the use of experiential avoidance was a risk factor for mental health while the rest three strategies played protective roles in mental health.

Positive emotions at T1 were positively related to the usage of experiential avoidance, perspective-taking, soothing, the willingness to retweet, and the saving behavior of humorous memes at T2. Life satisfaction at T1 was positively related to the use of enhancing positive emotions, social modeling, and the willingness to retweet at T2. The loneliness at T1 was negatively related to the use of all the interpersonal emotion regulation strategies except perspective-taking. These results suggested that certain psychological states promote the different use of emotion regulation strategies.

### 3.4 Cluster results of emotion regulation strategies at T1

**Figure 1:**
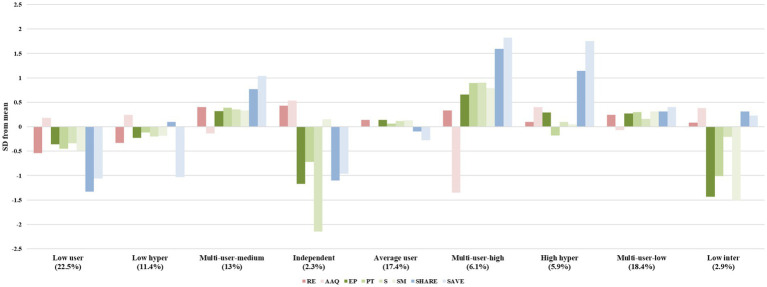
Multi-level emotion regulation profiles (Standardized deviations from the means). Bars represent the standardized deviation from the mean of the ER strategies of each profile. The number in parentheses represent the profile size (percentage of occasions about a profile); RE, cognitive reappraisal; AAQ, experiential avoidance; EP, enhancing positive effect; PT, perspective taking; S, soothing; SM, social modeling; WILL, retweeting willingness; SAVE, save behavior.

Control variables only affected a few emotion regulation strategies scores and clustering analysis is more powerful with larger sample sizes, so we elected to regress out these effects rather than conduct the analysis separately by age, gender, and risk level as other researchers do (Xie et al., 2022). The results indicated the presence of nine groups displaying different profiles of emotion regulation strategies (BIC = −30418.75; ICL = −30559.66). To enhance profile interpretability, we computed the standardized deviation from the means for each strategy within each profile (Figure 1). The bar chart of the average scores can be found in Supplementary Figure S1.

In profile 1 (22.5%, *n* = 201), all emotion regulation strategies except experiential avoidance deviated negatively (implying they were used below the mean level), so we labeled this profile “low user.” In profile 2, humorous meme saving deviated negatively, and the remaining strategies approached the mean (11.4%, *n* = 102), so we labeled it as “low hyper.” Profile 3 (13%, *n* = 116), 6 (6.1%, *n* = 55), and 8 (18.4%, *n* = 165) had similar patterns but to different degrees, in which all strategies except experiential avoidance deviated positively, so we labeled them as “multi-user.” In profile 4 (2.3%, *n* = 21), intra-personal strategies deviated positively, and the remaining strategies except social modeling, which approached the mean, deviated negatively, so we labeled it “independent.” In profile 5 (17.4%, *n* = 156), none of the ER strategies deviated noticeably from the mean; therefore, this profile was named “average user.” In profile 7 (5.9%, *n* = 53), the hyper-personal strategies deviated positively from the mean and the remaining approached the mean, so we labeled it as “high hyper.” In profile 9 (2.9%, *n* = 26), the interpersonal strategies deviated negatively and the remaining approached the mean, so we labeled it as “low inter.”

**Figure 2:**
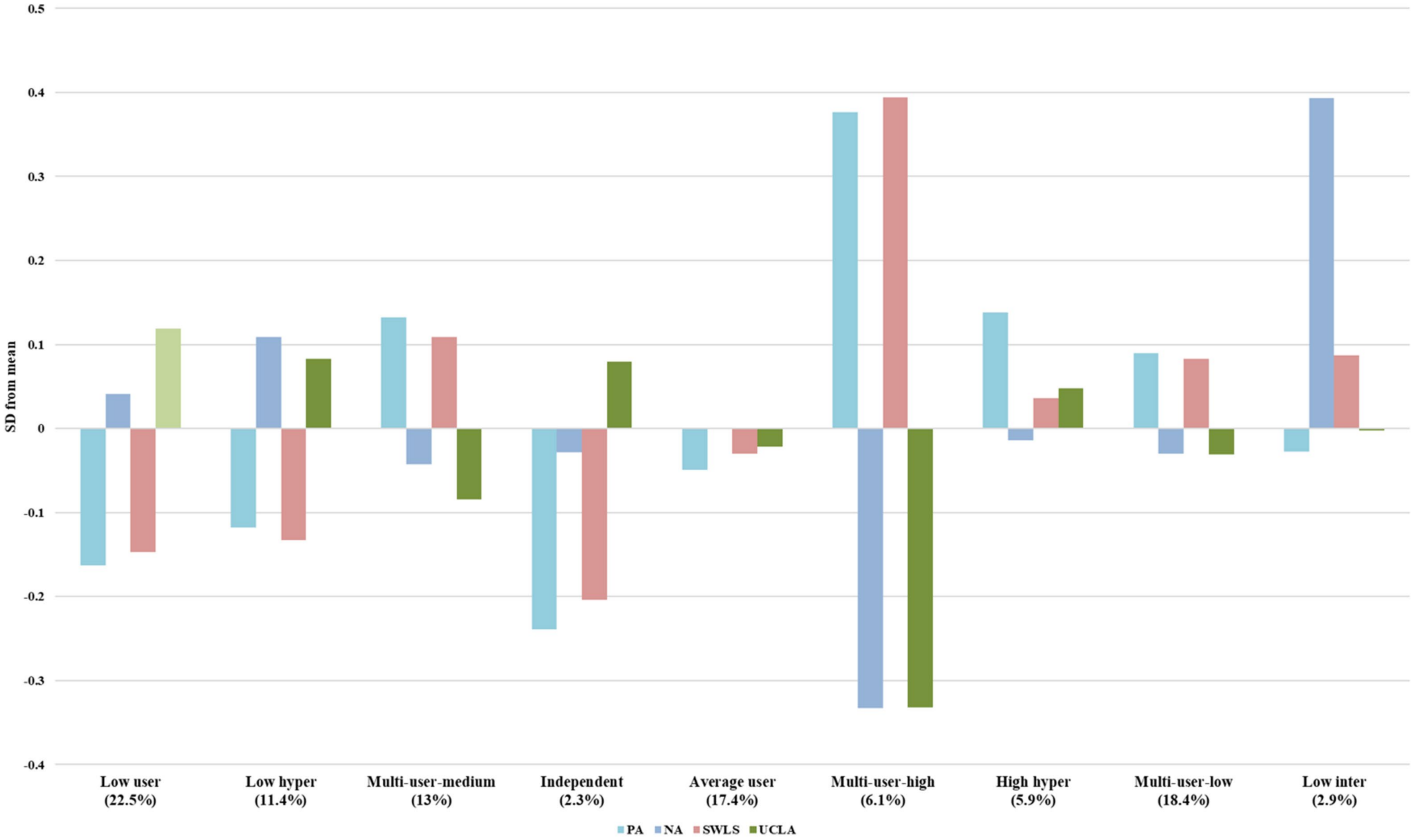
Mental health indicators of different profiles (Standardized deviations from the means). Bars represent the standardized deviation from the mean of the ER strategies of each profile. The number in parentheses represent the profile size (percentage of occasions about a profile); NA, negative emotion; PA, positive emotion; SWLS, life satisfaction; UCLA, loneliness.

We then compared emotion and mental health scores based on the cluster analysis results, and there were significant main effects of the group at T1 on the emotion and mental health scores at two measure points (*p*s < 0.001). *Post-hoc* comparisons revealed that at T1 positive emotions were highest in the “multi-user-high” group, negative emotions were lowest in the “multi-use-high” groups, and the highest in the “low inter” group. Life satisfaction was the highest in the “multi-user-high” group, and loneliness was the lowest in the “multi-user-high” group. There were similar patterns at T2. We also computed the standardized deviation from the means for each mental health indicator within each profile at T1 (Figure 2). The bar chart of the average scores can be found in Supplementary Figure S2. It is not hard to notice that the “multi-user” groups displayed better psychological states, with positive emotions and life satisfaction deviated positively and negative emotions and loneliness deviated negatively, and the “low user” group and the “low hyper” groups displayed the opposite outcomes from the “multi-user” groups. The “independent” group was characterized by negatively deviated positive mental health indicators (positive emotions and life satisfaction) and positively deviated loneliness. These findings indicated that only individuals who use multi-level strategies can maintain mental health when facing critical public events.

We further compared the differences in the use of emotion regulation strategies between the “multi-user” groups. Although the three “multi-user” groups had similar patterns, the mental health in the “multi-user-high” group was significantly better than the others. Compared to the “multi-user-low” group and the “multi-user-medium” group, the use of experiential avoidance was lower (*p*s < 0.001), and the use of soothing and humorous meme saving was higher (*p*s < 0.001) in the “multi-user high” group. These results were in line with the results in cross-lag analysis, which indicated that the use of experiential avoidance was a risk factor for mental health while humorous-meme-saving played a protective role in mental health.

## 4 Discussion

The current study examined emotion regulation strategies from a holistic and multilevel perspective. It evaluated the impact of the use of multi-level strategies on mental health in the context of COVID-19, and investigated existing emotion regulation profiles, finding that individuals who use all three levels of emotion regulation strategies (the multi-user) are better able to maintain mental health. These findings confirmed the importance of flexibility in emotion regulation for well-being, both from the perspective of the diversity and specific make-up of individuals’ ER repertoires (Grommisch et al., 2020).

### 4.1 The impacts of emotion regulation strategies on mental health

Cross-lagged analysis on the intrapersonal level strategies indicated that cognitive reappraisal at T1 was positively related to positive emotion at T2, implying that cognitive reappraisal is an effective affect-improving strategy, as found in previous studies (Aldao et al., 2010). Experiential avoidance was reciprocally positively related to negative emotions, confirming that experiential avoidance is a risk factor for mental health (Akbari et al., 2022). This reciprocal relationship raises the question of why people continue to use this strategy after experiencing negative feelings following its use. It may be explained by individuals’ motives for regulating emotions and affective forecasting errors, the ability to predict future emotions (Pilin, 2020). Avoidance of negative emotions was both consistent with the hedonic motive and utility motive (Tamir et al., 2007), but individuals may have a false expectation of its effect, which leads them to use experiential avoidance again, resulting in increased negative emotions (Akbari et al., 2022).

Regarding interpersonal-level strategies, results showed that perspective-taking was reciprocally positively related to life satisfaction. Research has demonstrated that individuals high in IER tendency are more socially connected (Williams et al., 2018), so perspective-taking may contribute to the development of supportive relationships, which benefit life satisfaction. Furthermore, perspective-taking refers to using others to remind oneself not to worry and that others have it worse, which involves talking to others and the modification of maladaptive appraisals and beliefs, may result in increasing their life satisfaction.

The study also found that humorous-meme-saving behavior was reciprocally positively related to positive emotions, suggesting that it is a protective factor for mental health. This finding is consistent with research on humor strategy, which indicates that humor strategy can effectively upregulate positive emotions and has a good intervention effect in depressed people and elderly people (Braniecka et al., 2022). Meanwhile, humor, also serves as a frequent and highly valued element of online communication, thus flourishing on social media (Laineste and Voolaid, 2017). This means although regulating emotions with digital technologies is a ‘double-edged sword’ (Erfani and Abedin, 2018; Smith et al., 2022), it remains a valuable channel for emotion regulation when it is related to positive content, as we found in our study.

### 4.2 The emotion regulation profiles promoting mental health

Cluster analysis suggested that there were 9 profiles with different usage preferences, which vary in terms of mental health indicators. Compared the mental indicators of individuals who primarily use intrapersonal strategies versus those who use a combination of intrapersonal and hyper-personal strategies, we found that the former exhibited high levels of loneliness and low levels of positive emotion and life satisfaction while the latter featured high negative emotions, high life satisfaction and average loneliness. This may indicate that intrapersonal strategies were effectively down-regulating negative emotions (Lepore et al., 2004) while associated with high loneliness, and hyper-personal strategies had a counter effect on loneliness since they helped to strengthen social connection (Hamilton et al., 2023) while having the risk of negative contagion (Prikhidko et al., 2020). It should be noted that people who balanced use of strategies at all three levels tended to be associated with greater positive emotions and life satisfaction while with lower negative emotions and loneliness, with the “multi-user-high” group significantly better than the others. This finding aligns with previous studies, indicating that frequent use of multiple strategies represents higher flexibility of emotion regulation, which is more adaptive for well-being (Grommisch et al., 2020).

Based on the result mentioned above, this may be attributed to the fact that different levels of emotion strategies serve as different resources and compensate for each other, allowing individuals to better cope with critical social events (Troy et al., 2013). For example, interpersonal emotion regulation is closely associated with the formation of intimate relationships that serve as relational resources to help people better cope with stressors (Schoebi and Randall, 2015), at the same time digital technologies provide more strategy choices that can be deployed with less effort (Smith et al., 2022). That is, the balanced use of strategies at all three levels expands solutions of emotion regulation, and improves the overall effectiveness of emotion regulation.

### 4.3 Implications and limitations

This study offers new insights into the relationship between emotion regulation and mental health. We examined the use of emotion regulation strategies from a multilevel perspective in the context of a real-life critical public event and re-examined the effectiveness of these strategies. These findings enrich the existing research on emotion regulation in mental health. Moreover, these findings provide empirical evidence of how individuals can maintain mental health when confronted with critical public events. The current study has identified the profile of emotion regulation strategies that can help individuals cope effectively with major social events, encouraging people to actively use strategies at all three levels.

We must acknowledge several limitations in this research. Firstly, the participants were only from China, and the dynamic zero-COVID policy in China stopped the spread of the virus efficiently, so more participants lived in low-risk areas which may potentially influence the outcomes of the study. Secondly, it is important to acknowledge the limitations related to the cross-sectional design of the study. This design hinders the ability to definitively establish cause-effect relationships, and the complexity of the model made it challenging to enforce strict invariance. Therefore, readers should interpret the findings with caution, and it would be ideal for future research to launch longitude studies with more measurement points and observe the changes. Thirdly, only self-report measures were used in this research, and the strategies for hyper-personal emotion regulation are not yet standardized in emotion research. Future studies should incorporate more objective measures. Finally, this study focused on a relatively longer time episode, but emotion regulation is a dynamic process, future research should opt for new methods such as ESM approaches to reflect the temporal aspects of emotion regulation, or employ new technologies such as virtual reality to explore emotion regulation in a higher ecological validity by simulating real-life settings (Colombo et al., 2019).

## 5 Conclusion

Critical public events often significantly impact people’s emotions and mental health, and ER strategies such as perspective-taking and humorous-meme-saving serve as effective mental health buffers in the face of critical public events, as evidenced by increased positive emotions and life satisfaction. In contrast, experiential avoidance was a risk factor positively associated with negative emotions. In Addition, the combination of high use of perspective-taking and humorous-meme-saving and low use of experiential avoidance predicts a better psychological state, characterized by high levels of positive emotions and life satisfaction and low levels of negative emotions and loneliness. These findings identify the profile that is protective for mental health during critical public events and confirm that emotion regulation flexibility is important for maintaining well-being.

## Data availability statement

The original contributions presented in the study are included in the article/Supplementary material, further inquiries can be directed to the corresponding author.

## Ethics statement

The studies involving humans were approved by the ethics committee of Sichuan Normal University. The studies were conducted in accordance with the local legislation and institutional requirements. The participants provided their written informed consent to participate in this study.

## Author contributions

LZ: Formal analysis, Investigation, Software, Visualization, Writing – original draft. JYa: Conceptualization, Funding acquisition, Project administration, Resources, Supervision, Writing – review & editing. JYu: Conceptualization, Funding acquisition, Resources, Supervision, Writing – review & editing.

## Footnotes

^1^Low-risk areas refer to areas other than medium and high-risk areas in the counties (cities, districts, banners) where medium and high-risk areas are located.
