# Supplementary material for: The use of multilevel emotion regulation strategies in the context of critical public events: the more the better?
Source: Front Psychol. 2024 Jul 15;15:1403308. doi: 10.3389/fpsyg.2024.1403308 (PMC11285105; doi:10.3389/fpsyg.2024.1403308)
Supplement: Supplementary file 1 [file Table_1.DOCX]

| **Table S1. Pearson correlations between variables** | | | | | | | | | | | | | | | | | | | | | | | | |
| --- | --- | --- | --- | --- | --- | --- | --- | --- | --- | --- | --- | --- | --- | --- | --- | --- | --- | --- | --- | --- | --- | --- | --- | --- |
| **Variable** | **T1NA** | **T1PA** | **T1SWLS** | **T1UCLA** | **T1RE** | **T1AAQ** | **T1EP** | **T1PT** | **T1S** | **T1SM** | **T1WILL** | **T1SAVE** | **T2NA** | **T2PA** | **T2SWLS** | **T2UCLA** | **T2RE** | **T2AAQ** | **T2EP** | **T2PT** | **T2S** | **T2SM** | **T2WILL** | **T2SAVE** |
| 1. T1NA | — |  |  |  |  |  |  |  |  |  |  |  |  |  |  |  |  |  |  |  |  |  |  |  |
| 2.T1 PA | -0.40^***^ | — |  |  |  |  |  |  |  |  |  |  |  |  |  |  |  |  |  |  |  |  |  |  |
| 3. T1SWLS | -0.42^***^ | 0.64^***^ | — |  |  |  |  |  |  |  |  |  |  |  |  |  |  |  |  |  |  |  |  |  |
| 4. T1UCLA | 0.45^***^ | -0.35^***^ | -0.47^***^ | — |  |  |  |  |  |  |  |  |  |  |  |  |  |  |  |  |  |  |  |  |
| 5. T1RE | -0.18^***^ | 0.29^***^ | 0.34^***^ | -0.34^***^ | — |  |  |  |  |  |  |  |  |  |  |  |  |  |  |  |  |  |  |  |
| 6. T1AAQ | 0.48^***^ | -0.34^***^ | -0.46^***^ | 0.62^***^ | -0.26^***^ | — |  |  |  |  |  |  |  |  |  |  |  |  |  |  |  |  |  |  |
| 7. T1EP | -0.22^***^ | 0.34^***^ | 0.34^***^ | -0.31^***^ | 0.48^***^ | -0.28^***^ | — |  |  |  |  |  |  |  |  |  |  |  |  |  |  |  |  |  |
| 8. T1PT | -0.15^***^ | 0.32^***^ | 0.33^***^ | -0.20^***^ | 0.44^***^ | -0.19^***^ | 0.57^***^ | — |  |  |  |  |  |  |  |  |  |  |  |  |  |  |  |  |
| 9. T1S | -0.11^**^ | 0.30^***^ | 0.31^***^ | -0.27^***^ | 0.40^***^ | -0.19^***^ | 0.64^***^ | 0.57^***^ | — |  |  |  |  |  |  |  |  |  |  |  |  |  |  |  |
| 10. T1SM | -0.18^***^ | 0.32^***^ | 0.31^***^ | -0.23^***^ | 0.54^***^ | -0.18^***^ | 0.64^***^ | 0.67^***^ | 0.63^***^ | — |  |  |  |  |  |  |  |  |  |  |  |  |  |  |
| 11. T1WILL | -0.08^*^ | 0.33^***^ | 0.32^***^ | -0.22^***^ | 0.26^***^ | -0.16^***^ | 0.31^***^ | 0.31^***^ | 0.36^***^ | 0.31^***^ | — |  |  |  |  |  |  |  |  |  |  |  |  |  |
| 12. T1SAVE | -0.16^***^ | 0.41^***^ | 0.40^***^ | -0.25^***^ | 0.28^***^ | -0.22^***^ | 0.30^***^ | 0.30^***^ | 0.33^***^ | 0.30^***^ | 0.79^***^ | — |  |  |  |  |  |  |  |  |  |  |  |  |
| 13. T2NA | 0.64^***^ | -0.37^***^ | -0.39^***^ | 0.39^***^ | -0.17^***^ | 0.43^***^ | -0.18^***^ | -0.15^***^ | -0.13^***^ | -0.15^***^ | -0.11^**^ | -0.17^***^ | — |  |  |  |  |  |  |  |  |  |  |  |
| 14. T2PA | -0.32^***^ | 0.69^***^ | 0.55^***^ | -0.39^***^ | 0.34^***^ | -0.33^***^ | 0.34^***^ | 0.33^***^ | 0.29^***^ | 0.34^***^ | 0.33^***^ | 0.40^***^ | -0.38^***^ | — |  |  |  |  |  |  |  |  |  |  |
| 15. T2SWLS | -0.36^***^ | 0.57^***^ | 0.77^***^ | -0.46^***^ | 0.32^***^ | -0.47^***^ | 0.34^***^ | 0.34^***^ | 0.32^***^ | 0.30^***^ | 0.34^***^ | 0.39^***^ | -0.41^***^ | 0.65^***^ | — |  |  |  |  |  |  |  |  |  |
| 16. T2UCLA | 0.42^***^ | -0.39^***^ | -0.44^***^ | 0.71^***^ | -0.32^***^ | 0.53^***^ | -0.32^***^ | -0.24^***^ | -0.30^***^ | -0.28^***^ | -0.24^***^ | -0.31^***^ | 0.50^***^ | -0.47^***^ | -0.51^***^ | — |  |  |  |  |  |  |  |  |
| 17. T2RE | -0.16^***^ | 0.29^***^ | 0.31^***^ | -0.29^***^ | 0.64^***^ | -0.24^***^ | 0.34^***^ | 0.35^***^ | 0.30^***^ | 0.41^***^ | 0.25^***^ | 0.28^***^ | -0.24^***^ | 0.40^***^ | 0.42^***^ | -0.40^***^ | — |  |  |  |  |  |  |  |
| 18. T2AAQ | 0.46^***^ | -0.35^***^ | -0.41^***^ | 0.49^***^ | -0.22^***^ | 0.74^***^ | -0.32^***^ | -0.22^***^ | -0.23^***^ | -0.25^***^ | -0.17^***^ | -0.24^***^ | 0.51^***^ | -0.40^***^ | -0.50^***^ | 0.60^***^ | -0.30^***^ | — |  |  |  |  |  |  |
| 19. T2EP | -0.23^***^ | 0.35^***^ | 0.37^***^ | -0.31^***^ | 0.40^***^ | -0.29^***^ | 0.69^***^ | 0.48^***^ | 0.53^***^ | 0.49^***^ | 0.37^***^ | 0.38^***^ | -0.27^***^ | 0.42^***^ | 0.44^***^ | -0.41^***^ | 0.47^***^ | -0.39^***^ | — |  |  |  |  |  |
| 20. T2PT | -0.14^***^ | 0.32^***^ | 0.34^***^ | -0.23^***^ | 0.40^***^ | -0.25^***^ | 0.44^***^ | 0.64^***^ | 0.46^***^ | 0.52^***^ | 0.36^***^ | 0.36^***^ | -0.20^***^ | 0.38^***^ | 0.43^***^ | -0.31^***^ | 0.49^***^ | -0.27^***^ | 0.59^***^ | — |  |  |  |  |
| 21. T2S | -0.12^***^ | 0.33^***^ | 0.32^***^ | -0.27^***^ | 0.40^***^ | -0.20^***^ | 0.53^***^ | 0.50^***^ | 0.71^***^ | 0.55^***^ | 0.33^***^ | 0.34^***^ | -0.19^***^ | 0.42^***^ | 0.41^***^ | -0.36^***^ | 0.46^***^ | -0.26^***^ | 0.68^***^ | 0.65^***^ | — |  |  |  |
| 22. T2SM | -0.15^***^ | 0.32^***^ | 0.33^***^ | -0.27^***^ | 0.51^***^ | -0.22^***^ | 0.50^***^ | 0.54^***^ | 0.51^***^ | 0.66^***^ | 0.36^***^ | 0.37^***^ | -0.19^***^ | 0.42^***^ | 0.42^***^ | -0.35^***^ | 0.57^***^ | -0.27^***^ | 0.63^***^ | 0.71^***^ | 0.72^***^ | — |  |  |
| 23. T2WILL | -0.10^**^ | 0.34^***^ | 0.34^***^ | -0.21^***^ | 0.25^***^ | -0.15^***^ | 0.26^***^ | 0.27^***^ | 0.30^***^ | 0.26^***^ | 0.79^***^ | 0.72^***^ | -0.14^***^ | 0.38^***^ | 0.37^***^ | -0.27^***^ | 0.28^***^ | -0.19^***^ | 0.38^***^ | 0.34^***^ | 0.34^***^ | 0.35^***^ | — |  |
| 24. T2SAVE | -0.15^***^ | 0.42^***^ | 0.39^***^ | -0.23^***^ | 0.25^***^ | -0.20^***^ | 0.26^***^ | 0.28^***^ | 0.29^***^ | 0.27^***^ | 0.69^***^ | 0.84^***^ | -0.17^***^ | 0.45^***^ | 0.42^***^ | -0.31^***^ | 0.29^***^ | -0.25^***^ | 0.37^***^ | 0.35^***^ | 0.34^***^ | 0.35^***^ | 0.82^***^ | — |
| *Note*: *p < .05, **p < .01, ***p< .001. NA=negative emotion; PA=positive emotion; SWLS=life satisfaction; UCLA=loneliness; RE=cognitive reappraisal; AAQ=experiential avoidance; EP=enhancing positive affect; PT=perspective taking; S=soothing; SM=social modeling; WILL=retweeting willingness; SAVE=save behavior | | | | | | | | | | | | | | | | | | | | | | | | |
