# Supplementary material for: The use of multilevel emotion regulation strategies in the context of critical public events: the more the better?
Source: Front Psychol. 2024 Jul 15;15:1403308. doi: 10.3389/fpsyg.2024.1403308 (PMC11285105; doi:10.3389/fpsyg.2024.1403308)
Supplement: Supplementary file 2 [file Table_2.DOCX]

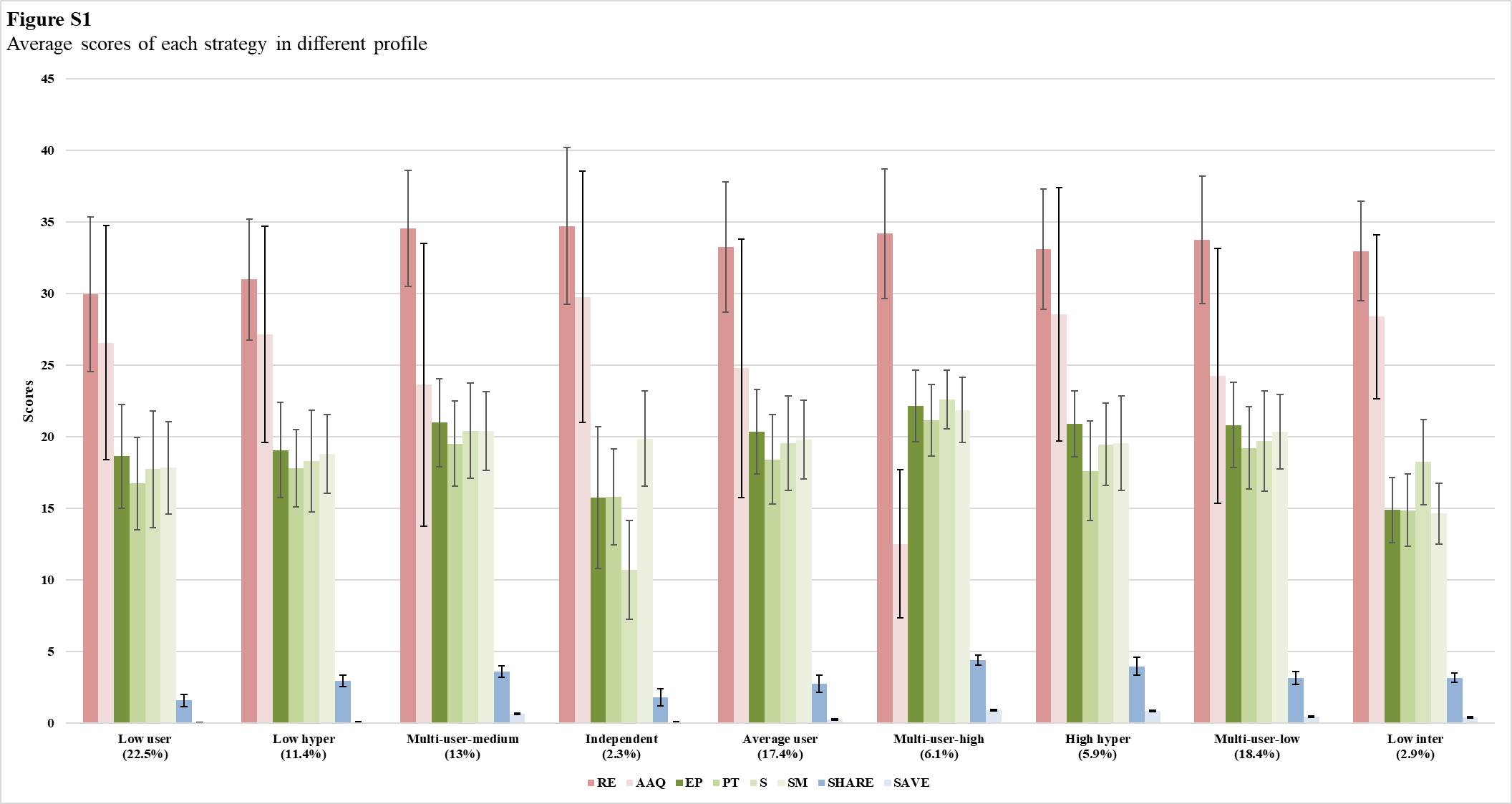
Note. Bars represent the average scores of the ER strategies of each profile. The numbers in parentheses represent the profile size (percentage of occasions about a profile); RE=cognitive reappraisal; AAQ=experiential avoidance; EP=enhancing positive affect; PT=perspective taking; S=soothing; SM=social modeling; WILL=retweeting willingness; SAVE=save behavior. Error bar=std.


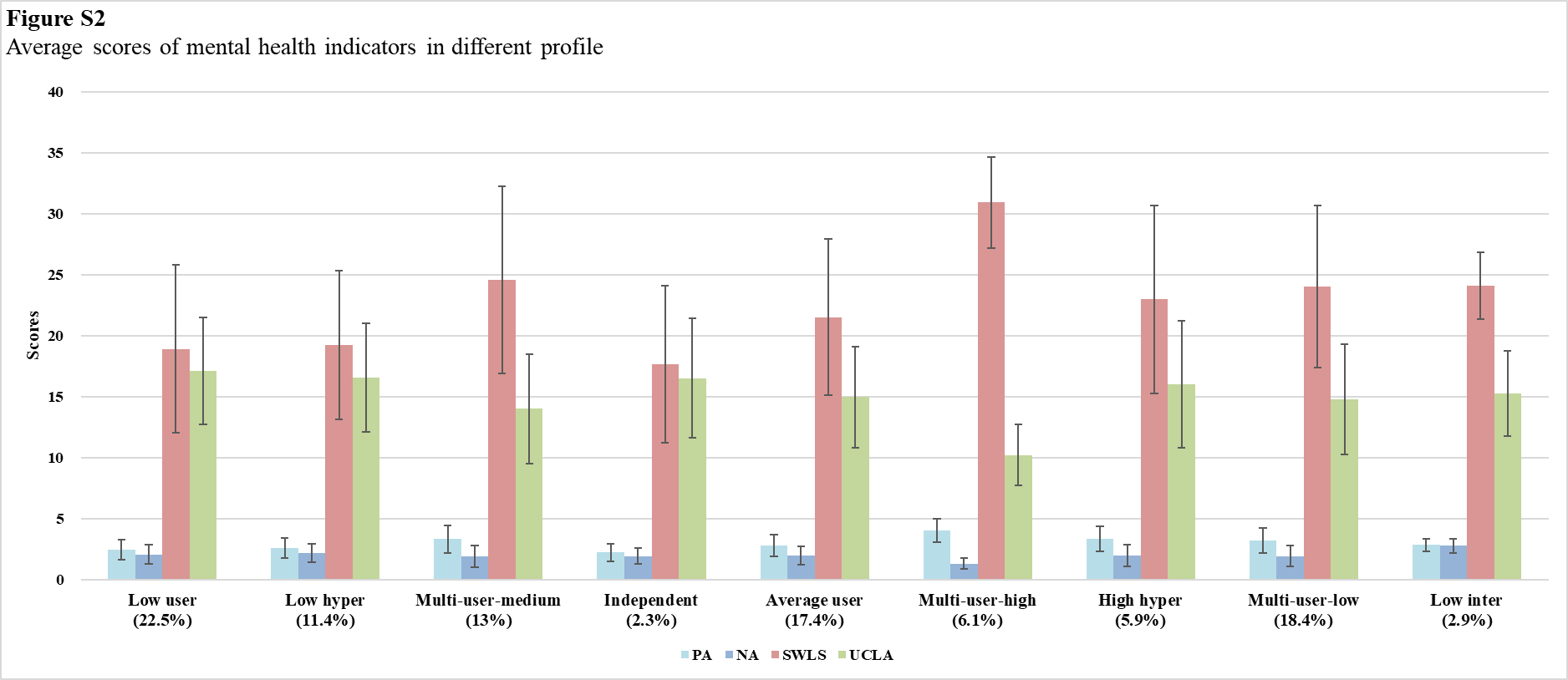
Note. Bars represent the average scores of the mental indicators of each profile. The numbers in parentheses represent the profile size (percentage of occasions about a profile); NA=negative emotion; PA=positive emotion; SWLS=life satisfaction; UCLA=loneliness. Error bar=std.
